# Parkinson's Disease: Current Treatment Modalities and Emerging Therapies

**DOI:** 10.7759/cureus.75647

**Published:** 2024-12-13

**Authors:** Shabab Alotaibi, Lujain Alfayez, Mohammed Alkhudhair

**Affiliations:** 1 Neurology, Movement Disorder and Neuromodulation, King Saud Medical City, Riyadh, SAU; 2 Neurology, Neurology Center, Prince Sultan Military Medical City, Riyadh, SAU; 3 Neurology, King Faisal Specialist Hospital and Research Centre, Riyadh, SAU

**Keywords:** deep brain stimulation, movement disorders and tremors, parkinson' s disease, parkinson’s management, tremor

## Abstract

Herein, we review the literature on Parkinson’s disease (PD) management and summarize the progress in medical, surgical, and assisted therapeutic modalities for motor and non-motor symptoms. A thorough search strategy was implemented to retrieve all relevant articles and identify the best evidence from different databases including Scopus, PubMed, Google Scholar, the Cochrane Database of Systematic Reviews, and Evidence-Based Medicine reviews from the International Parkinson and Movement Disorder Society. Multiple terms, such as Parkinson, tremor, predominant, Parkinson management, deep brain stimulation, LCIG, ablative surgery for PD, medical management of PD, and assistive devices for PD, were used for screening. A total of 160 articles were gathered; irrelevant papers and older articles were excluded. After initial exclusion, we had 140 articles to review from 1980 to 2022. Five articles were found to be duplicated, and another five articles were excluded as they did not have additional information on management that could be used in this research paper. We found that management options and assistive devices for PD are improving, with new therapeutic options emerging every year. Medical therapy is the most common therapy as it corrects dopamine deficiency which is the main factor implicated in PD; other surgical treatment options are available in cases of chronic progressive disease course. This article adds significant value to the literature as it includes the history and the role of most Parkinson’s disease management options.

## Introduction and background

Parkinson’s disease (PD) is one of the most prevalent chronic progressive neurological disorders and the second most common neurodegenerative disorder, affecting > 1.5% of cases worldwide in populations older than 65 years [1]. It usually occurs due to decreased dopamine production due to dopaminergic neuronal death in the midbrain's substantia nigra, leading to striatal dopamine deficiency, which is responsible for the motor symptoms of PD [2]. Exogenous toxins, inflammation, genetic alterations, and combinations of these variables are suggested mechanisms. PD is widely believed to result from an interaction between hereditary and environmental factors. This hypothesis states that cell death in nigral neurons is caused by an interaction between environmental conditions and genetic predisposition, resulting in mitochondrial respiratory failure and oxidative stress [3]. The median spiny neurons of the midbrain are the primary targets of PD, but additional losses of dopaminergic neurons and accumulation of Lewy bodies in the substantia nigra pars compacta (SNpc) affect normal physiological neuronal functioning. Additionally, GABAergic neuron excitability in PD is increased at the dopaminergic D2 receptor but decreased at the D1 receptor, indicating an imbalance between the direct and indirect pathways. During this time, stiffness and bradykinesia resurfaced. Upregulation of the alpha-synuclein gene causes the production of aberrant mutant alpha-synuclein protein, which aggregates to form Lewy bodies and neuritis, causes neurodegeneration, and manifests symptoms similar to those of PD. However, cellular malfunction and mortality are caused by oxidative stress and mitochondrial dysfunction [4].

**Figure 1:**
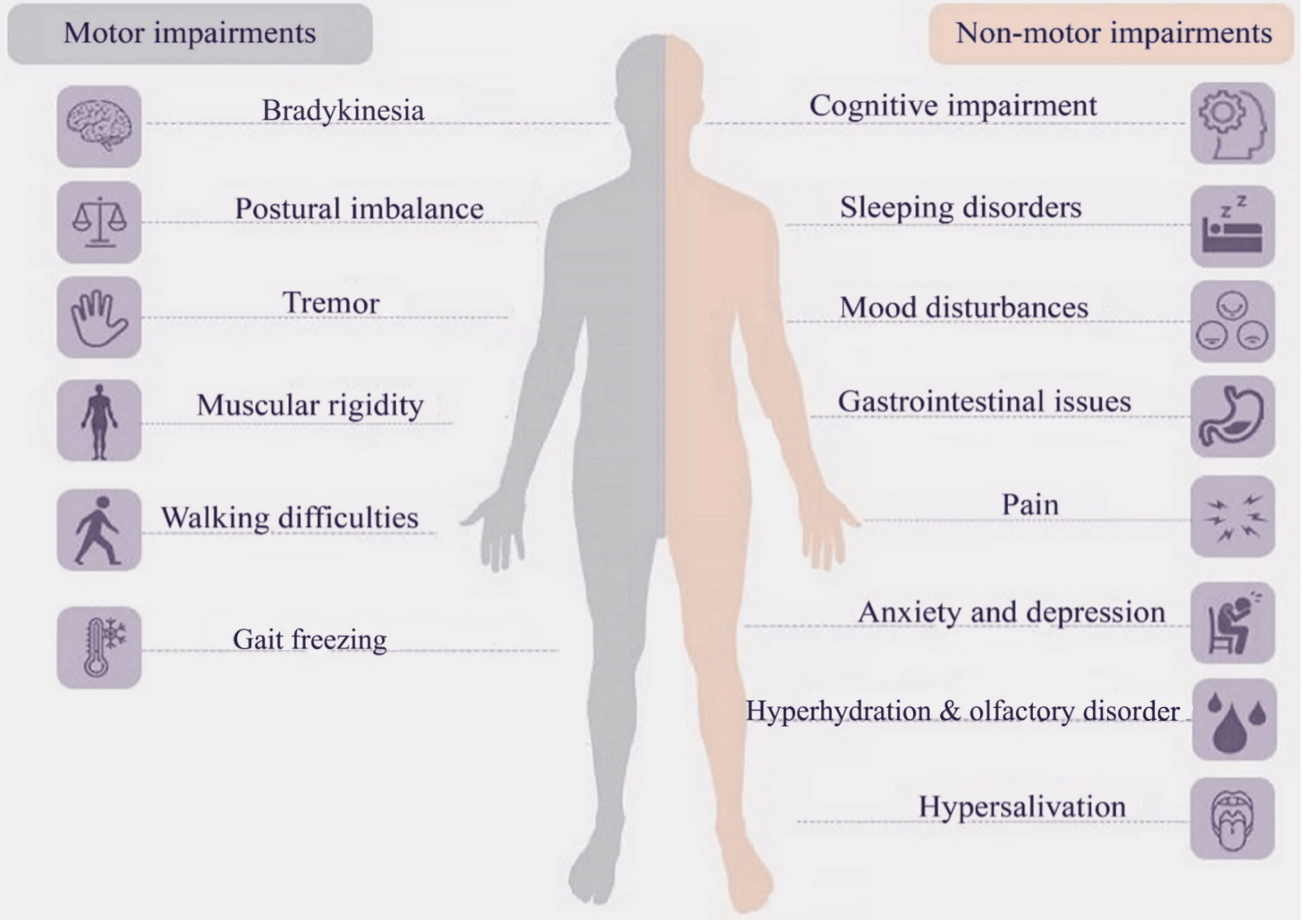
Parkinson's disease clinical manifestations

PD can be diagnosed based on the patient’s clinical features and exclusion of other possible causes of PD since there are no practical laboratory tests to diagnose PD (Figure 1).

Despite marked efforts to determine the cause of PD, its etiology and exact cause remain obscure, leading to treatment limitations [5].

The management of PD requires individual customization of treatment at all stages as the disease progresses [6]. The management options and assisted methodologies of PD are improving, with new therapeutic options emerging every year. While medical therapy is the most common therapy as it corrects dopamine deficiency, which is the main factor implicated in PD, other surgical treatment options are available in cases of a chronic progressive disease course [7]. Common medications include levodopa/carbidopa, carbidopa/levodopa/entacapone (Stalevo), dopamine receptor agonists (bromocriptine, ropinirole, pramipexole, apomorphine), selective monoamine oxidase-B inhibitors (selegiline and rasagiline), anticholinergic agents (muscarinic receptor antagonists: artane, Cogentin, Benadryl), and amantadine (Symmetrel) [7]. However, the current surgical options are ablative procedures (lesional therapy), dopaminergic medication infusion devices, and deep brain stimulation (DBS) [8].

**Figure 2:**
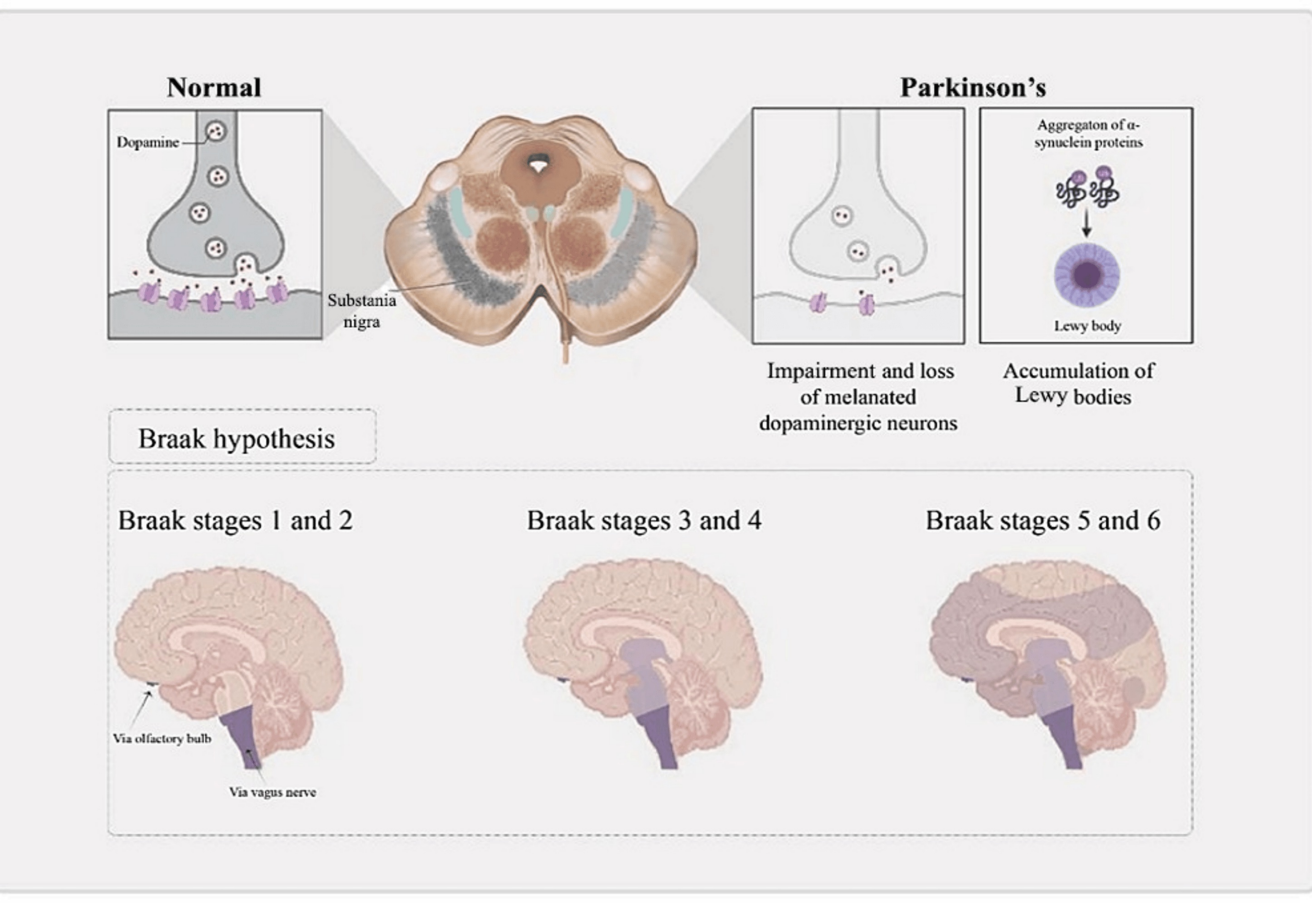
Parkinson's disease pathophysiology and Braak staging

Dopaminergic neuron loss in the substantia nigra is a hallmark of PD. Lewy bodies, neuronal inclusions primarily made up of a-synuclein protein aggregations, are pathological characteristics of PD. The Braak hypothesis is the most frequently used theory for explaining the neuropathological development of PD (Figure 2) [7].

According to this hypothesis, PD (stages 1 and 2) first manifests itself in the medulla and olfactory bulb. Rapid eye movement sleep behavior disorder (in which people lose their normal rapid eye movement sleep paralysis and have physical actions while sleeping) and impaired smell are symptoms that are linked to this early disease. The substantia nigra pars compacta and other midbrain and basal forebrain regions are affected by stage 3 and stage 4 disease. Motor symptoms of PD are associated with pathology in these regions. At this point, PD is often identified. The illness spreads to the cerebral cortex when PD advances, and cognitive impairment and hallucinations begin to develop. Protein aggregation in Parkinson's is linked to the death of cells that produce dopamine. Dopamine supplementation is the cornerstone of PD treatment. However, PD also affects other neurotransmitter systems, including serotonin, acetylcholine, norepinephrine, and acetylcholine systems (Table 1) [9]. This explains why some PD symptoms resist treatment with drugs based on dopamine. These neurotransmitter systems are the focus of emerging therapeutic strategies. This study aimed to explore the current treatments and emerging therapies for PD.

**Table 1:** Most commonly prescribed therapeutics for managing PD-related motor symptoms

Category	Name	Type/Administration	Useful for	Side effects
Dopamine like	Carbidopa/levodopa	Immediate release/controlled release/extended-release	Monotherapy or with levodopa/motor fluctuations	Hallucinations, nausea
		Jujenal gel infusion	Motor fluctuations	Abdominal pain, infections, nausea
	levodopa	Inhalation powder	Rescue motor fluctuations	Hallucinations, nausea, and cough
Dopamine agonists	Ropinirole/Pramipexole	Immediate release/extended release	Monotherapy or with levodopa/motor fluctuations	Hallucinations, nausea, and drowsiness
	Rotigotine	Patch	Monotherapy or with levodopa/motor fluctuations	Hallucinations, nausea, skin reactions, and drowsiness
	Apomorphine	Sublingual/injections	Rescue motor fluctuations	Nausea, site reactions
COMT inhibitors	Opicapone		Motor fluctuations	Dizziness, orthostatic hypotension
	Tolcapone			Hepatotoxicity, orange discoloration of urine, diarrhea, and nausea
	Entacapone			Orange discoloration of urine, diarrhea, and nausea
MAO-B inhibitors	Selegiline/Rasagiline		Monotherapy or with levodopa/motor fluctuations	Nausea, dizziness, insomnia
	Safinamide		Motor fluctuations	
Anti-glutamates	Amantadine	Immediate release/sustained release	Monotherapy or with levodopa/dyskinesias motor fluctuations	Hallucinations/pedal edema, constipation, livedo reticularis, and insomnia
Anticholinergics	Trihexyphenidyl/benztropine		Monotherapy or with levodopa (rest tremor)	Urinary retention, blurry vision, dry mouth, and cognitive impairment

## Review

Methods

**Figure 3:**
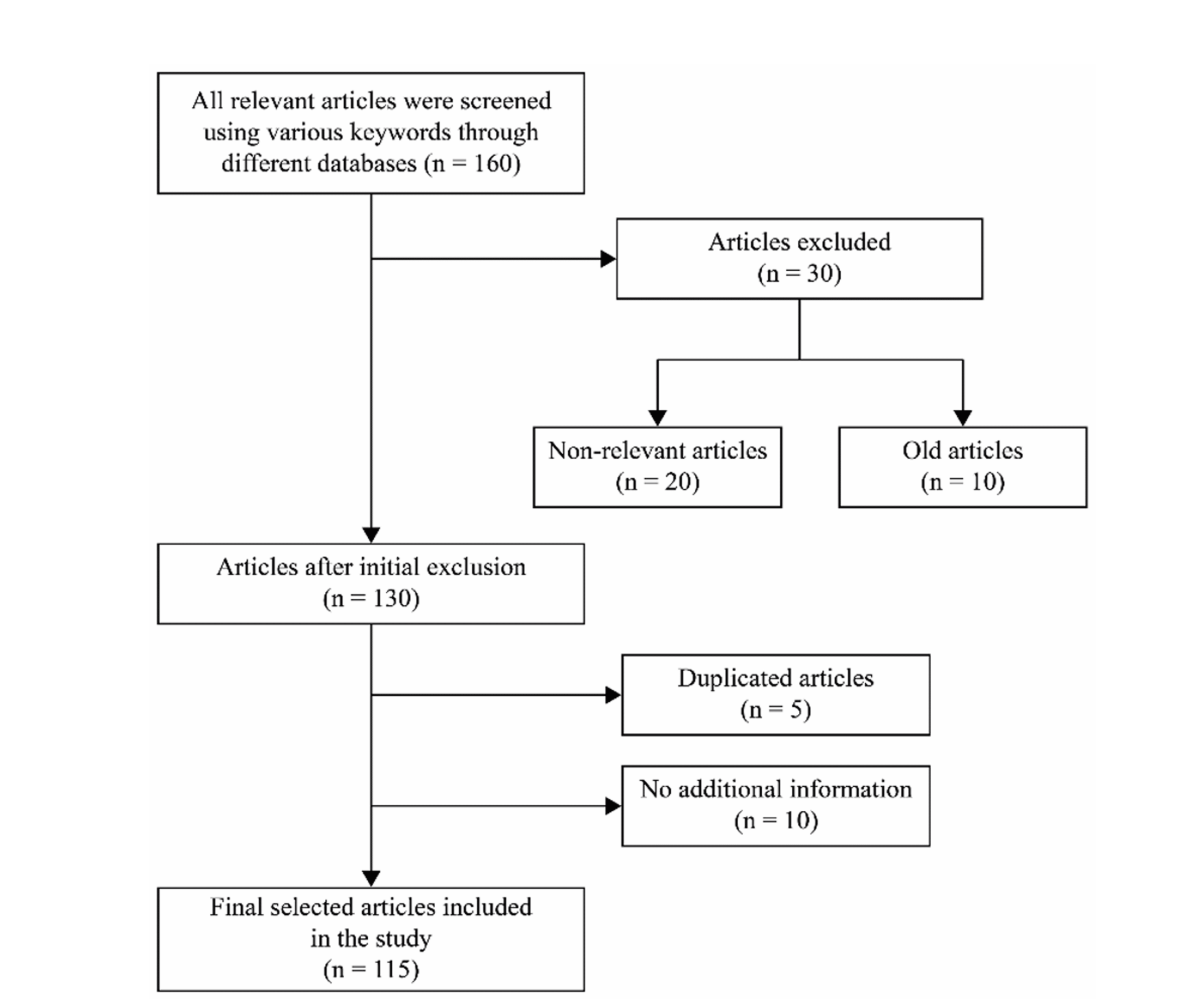
Flow diagram summarizing the steps involved in the literature search In the above figure, "old articles" refer to those published before 1980.

We conducted a thorough search strategy to find all relevant articles and identify the best evidence. First, all relevant literature was reviewed to gather all published data on PD diagnosis and management. Moreover, an extensive search was conducted using different databases, including Scopus, PubMed, Google Scholar, the Cochrane Database of Systematic Reviews, and Evidence-Based Medicine reviews from the International Parkinson and Movement Disorder Society. Multiple terms were screened, such as Parkinson, tremor, predominant, Parkinson management, DBS, LCIG, ablative surgery for PD, medical management of PD, and assistive devices for PD. A total of 160 articles were gathered, and irrelevant papers and old articles were excluded; after the initial exclusion, there were 140 articles to review from 1980 to 2022. Five articles were duplicated, and the other five were excluded as they did not have additional management information that could be used in this research paper. A flow diagram summarizing the steps involved in the literature search is presented in Figure 3.

Medical therapeutics

Levodopa/Carbidopa

In 1961, Hornykiewicz was the first to introduce the use of levodopa to increase dopamine levels and improve PD symptoms. In 1974, carbidopa was added to overcome adverse gastrointestinal reactions associated with levodopa usage [10].

Mechanism of action: The mechanism of action of levodopa works by providing exogenous dopamine and activating central dopamine receptors in the nigrostriatal system, which improves PD symptoms. However, the major advantage of levodopa is that it can be used in different stages of PD [11]. This drug is usually administered with decarboxylase, catechol-O-methyl transferase (COMT), or aromatic-L-amino-acid decarboxylase (AADC) inhibitors [11]. This prevents the peripheral metabolism of dopamine by COMT and AADC. Levodopa is absorbed from the gastrointestinal tract and passes through the blood-brain barrier (BBB). However, it also has a short half-life. Therefore, it should be administered multiple times to prevent plasma level fluctuations [12, 13].

Safety and efficacy*: *Several studies have shown that levodopa is the most potent treatment modality for managing the motor symptoms of PD. Further, this drug has the best efficacy in these patients because of the lack of dopaminergic activity. The efficacy of levodopa has been shown to exceed that of other comparable PD treatment modalities, including dopamine agonists, anticholinergics, monoamine oxidase-B (MAO-B) inhibitors, and amantadine, in mobility outcomes and quality-of-life measures. However, the administration of this treatment modality does not usually affect disease progression [14-16]. There are multiple routes of administration for levodopa, such as inhaled levodopa, subcutaneous levodopa, and intestinal fusion levodopa. Some side effects of levodopa have been reported, including sleep, psychological disturbances, and gastrointestinal adverse events such as nausea and vomiting. Autonomic side effects have also been reported, including orthostatic hypotension, dizziness, and daytime somnolence [17].

The fluctuation of pharmacodynamic response to LD between wearing off and LD-induced dyskinesias leads to a narrower therapeutic window with a higher risk of overdosage and underdosage [12]. Furthermore, the disease is usually associated with a characteristic phenomenon of honeymoon wear as the clinical response continues while dyskinesia and motor fluctuations wear off after the initial period [14]. Dyskinesia and motor fluctuations are other reported adverse events of levodopa after prolonged usage that can significantly impact the quality of life in the long term [14, 18]. Overcoming these events can be achieved by adding dopamine agonists (such as pramipexole and ropinirole), changing the formulation of levodopa, changing the administration regimen, or adding amantadine. It has been further shown that combining other treatment modalities, such as COMT and MAO-B inhibitors, can also be useful in these situations [14, 19].

Dopamine Agonists

The first trial to evaluate the effects of bromocriptine versus levodopa was in the mid-1980s. Initially, the dopamine agonist was considered an adjuvant medication in late PD. Many research efforts have been conducted to delay the introduction of levodopa in patients, which has eventually led to the development of dopamine agonists as monotherapy, with a theory stating that the administration of dopamine agonists can delay the development of motor complications if administered before levodopa [20].

Mechanism of action (which receptor works on tremor): Dopamine agonists directly stimulate dopamine receptors in the brain by mimicking the behavior of neurotransmitters. Two types of dopamine agonists were reported: ergot and non-ergot. Ergot types include lisuride, pergolide, cabergoline, and bromocriptine. These modalities can work remarkably on D2-like receptors, including D2, 3, and 4 [21]. In contrast, pramipexole and ropinirole are non-ergot dopamine agonists. These modalities are highly selective and potent, explicitly acting on D2 receptors. Moreover, pramipexole has a remarkable affinity for D3 receptors [21, 22].

Efficacy and safety: Administering dopamine agonists as the initial treatment for three to five years in PD can reduce dyskinesia. The Movement Disorder Society debate is still ongoing on whether to start or delay the initiation of levodopa in young patients; however, the current acceptable approach is to combine dopamine agonists with levodopa as an initial treatment regimen, given that it has a better outcome in this context [14, 15, 23-26]. Moreover, the evidence of long-term outcomes requires further strengthening. Pulmonary fibrosis is frequently reported among patients receiving ergot types, and the frequency of adverse events with dopamine agonists is higher than that with levodopa because of their proven action on peripheral dopamine receptors. Day sleepiness is frequently reported in patients receiving non-ergot dopamine agonists; edema, orthostatic hypotension, and nausea are the most frequently reported side effects. Therefore, changing the formulation and medication type should be practiced according to the patient's reaction to these medications. Other reported adverse events include neuropsychiatric disorders such as dopaminergic dysregulation syndrome, dopamimetic-induced psychosis, and impulse control disorder [14, 27].

OMT Inhibitors

In 1998, the Food and Drug Administration (FDA) approved the first COMT inhibitor for the market, tolcapone; this class of drugs revealed marked symptomatic improvement in PD patients [28].

Mechanism of action: These treatment modalities inhibit the COMT enzyme, reducing catecholamine levels, including norepinephrine and dopamine degradation. The enzyme is widely found in peripheral and central regions, and COMT inhibitors mainly aim to reduce dopamine transmission and action through the BBB to the brain. However, these treatment modalities are not solely used and are administered in combination with levodopa to prevent methylation in the peripheral circulation. Studies have indicated that it can significantly reduce dyskinesia and enhance the bioavailability of levodopa [29].

Efficacy and safety: Evidence shows that although tolcapone is more efficacious than entacapone in managing PD symptoms, it is usually associated with an increased risk of hepatotoxicity [30]. Accordingly, it is no longer recommended for administration in many countries secondary to reporting three cases of fulminant hepatitis [27]. However, the drug can be used carefully with frequent monitoring of liver function. Some study results reported that reduction in the baseline dose of levodopa is significantly more prominent with tolcapone than with other COMT inhibitors [30].

The efficacies of opicapone and entacapone are similar. However, evidence is still scarce regarding the cost-effectiveness of opicapone [30-32]. No significant adverse events have been reported with COMT inhibitors, as most evidence shows they are well tolerated. However, some adverse events have been reported to occur secondary to dopaminergic stimulation. These events include nausea, hypotension, psychiatric events, diarrhea, and dyskinesia; thus, adjusting the dose of levodopa can significantly enhance associated dyskinesia. Additionally, brownish discoloration of the urine has also been reported. However, this is harmless to humans [30].

MAO-B Inhibitors

Selegiline was the first registered MAO-B inhibitor approved by the FDA in 1996. The main reason behind MAO-B use is to overcome LD neurotoxicity, and the DATATOP study was among the first to explore the potential neuroprotective effects of selegiline [17]. 

Mechanism of action: The main action of MAO-B inhibitors is to prolong the action of dopamine in DP by reducing its catabolism by MAO-B. However, it should be noted that selegiline is used as a monotherapy in early PD in mild cases within three to five years of diagnosis. It is also used in combination with other treatment modalities, such as levodopa. Based on the mechanism of action of these modalities, there is little evidence to suggest that MAO-B inhibitors can induce further neuroprotective effects in other diseases, such as Alzheimer's disease, by providing remarkable protective actions against neuronal functions and structures. The other reported mechanism for MAO-B inhibitors is the protection of mitochondrial functions, prolonged apoptosis-related neuronal cell death, and antioxidant effects, although the evidence is insufficient [33, 34].

Efficacy and safety: Rasagiline and selegiline are two major MAO-B inhibitors associated with favorable, irreversible, and selective effects. According to the DATATOP trial, the administration of MAO-B inhibitors can delay the need to administer levodopa for nine months. In addition, compared to placebo, patients receiving selegiline developed freezing of gait and on-off motor fluctuations less frequently. It has also been reported that these modalities can significantly reduce dyskinesia and enhance motor outcomes. This has been indicated in the ADAGIO and TEMPO trials with selegiline administration, especially with early compared to late administration, which can reduce the intensity of PD symptoms such as gait, postural instability, tremors, and bradykinesia [33].

Moreover, they can significantly decrease off-time and enhance motor function when used in combination with COMT inhibitors and dopamine agonists [33]. Research has demonstrated an increase in dyskinesia in patients on selegiline compared to placebo [17]. Overall, no significant adverse events were reported among patients receiving MAO-B inhibitors compared with those receiving placebo. Other reported side effects include postural hypotension, hallucinations, and depression among over 70 patients [33].

Others

Anticholinergics: Anticholinergics are important treatment modalities for PD because they can reduce the activation of acetylcholine by blocking cholinergic receptors. It is important to induce a balance between cholinergic and dopaminergic receptors in PD, as it is well established that lesions in the nigra striatum are usually observed in PD, significantly reducing dopamine concentrations in affected patients. This can significantly induce a state of cholinergic firing, indicating the need to reduce acetylcholine production and activation to create a balance. Therefore, administering anticholinergics can significantly reduce dyskinesia and tremors in patients with PD by acting on the M4 receptors [35, 36]. Anticholinergics such as trihexyphenidyl and benztropine are highly effective in treating PD symptoms and manifestations [37]. Still, it should be noted that these modalities are not helpful in patients with PD-related motor complications or PD prevention. Moreover, its long-term efficacy is not clearly established owing to the limited data available in this context. Nevertheless, these drugs can enhance motor performance in patients with PD compared with a placebo. In addition, they can be effectively used as monotherapy or in combination with other medications for PD [37].

Evidence shows that the administration of anticholinergics in patients with PD is usually associated with more adverse events than other treatment modalities. Some reported adverse events included nausea and vomiting, urinary retention, tachycardia, constipation, and blurred vision. These side effects usually develop secondary to the peripheral antimuscarinic effects of these modalities, and evidence has shown that some events might be severe and lead to the discontinuation of a study. Other events might impact the quality of life of elderly patients, including confusion, hallucinations, and difficulty memorizing [27, 36].

Propranolol: Tremor is a classic triad of PD and is usually referred to as tremor-at-rest. A study published in 2011 suggested a model for the pathophysiology of the classic tremor that either developed as a trigger from the basal ganglia or as a driving force from the cerebellothalamocortical network since the development of tremor is correlated with dopamine depletion in the pallidum and the magnitude of ongoing tremor was correlated with the cerebellar circuit activity demonstrated by functional magnetic resonance imaging [38]. The use of β-adrenergic antagonists has also been reported in the literature, with favorable outcomes for managing tremors in patients with PD. The administration of propranolol is particularly indicated when stress is a significant complaint; tremors worsen with anxiety, and a postural component is associated with the condition. Although this drug is widely used in clinical settings, evidence regarding its use is scarce in relevant studies in the literature [39]. However, it has been demonstrated that the long-term use of 160 mg is not associated with adverse events. However, the drug should be carefully administered to patients with advanced PD because of the risk of postural hypotension and bradycardia. Further evidence regarding the use of propranolol is needed. Generally, it has been demonstrated that treating tremors of PD using pharmacological approaches is challenging, highlighting the importance of surgical approaches [40].

Amantadine: Amantadine was discovered in the 1960s when PD patients in chronic care facilities noticed an improvement in symptoms after receiving amantadine as influenza prophylaxis [17].

The mechanism by which amantadine functions in PD is poorly understood. Evidence shows that the drug reduces dopamine reuptake and increases dopamine release by inhibiting cholinergic muscarinic receptors and N-methyl-D-aspartate (NMDA)-glutamate receptors. Similar to previous medications, the drug is not used as monotherapy in PD. Several clinical trials have demonstrated the efficacy of amantadine in reducing motor fluctuations in PD patients treated with levodopa [41]. However, it is usually used in combination with levodopa to reduce associated extrapyramidal manifestations and enhance dyskinesia [42].

It has been demonstrated that reducing dyskinesia events is most prominent with amantadine, among other pharmacological regimens [43]. This has been indicated in a recent meta-analysis, which showed that amantadine was significantly associated with the Unified Dyskinesia Rating Scale (UDysRS) total score and levodopa-associated dyskinesia [44]. However, the safety of amantadine remains controversial and warrants further investigation. However, evidence from in vivo and in vitro studies is controversial. It has been shown that no adverse events are usually found when receiving a dose < 15 mg/kg, although adverse events, such as neurotoxicity, stereotyping, and reduced memory and learning abilities, have been reported when the dose exceeds this limit. Moreover, a recent meta-analysis showed that orthostatic hypotension, prolonged QT interval, hallucinations, edema, constipation, and nausea were the most common adverse events (Table 1) [43, 45].

Levetiracetam: Levetiracetam is an anticonvulsant that can effectively treat dyskinesia in PD patients. It reduces neurotransmitter release by binding to the synaptic vesicle protein A2. The exact mechanism of action of this drug is not yet clearly understood. Previous preclinical studies have shown that it could be effectively combined with amantadine to treat levodopa-induced dyskinesia [44-46]. Unfortunately, data from observational studies show that levetiracetam cannot be well tolerated in patients with PD, unlike in patients with epilepsy.

Moreover, some randomized controlled trials have been conducted to investigate the impact of levetiracetam administration on reducing levodopa-induced dyskinesia. For instance, a study showed that the administration of 1 g/day of levetiracetam by patient-completed diaries in 38 patients with PD for five weeks significantly decreased the time with dyskinesia by 75 minutes (95% confidence interval: 3.31-12.4; p-value = 0.002). However, some adverse events have been reported, including somnolence and dizziness [47]. In contrast, other clinical trials have shown that this drug does not significantly reduce dyskinesia [48, 49]. A meta-analysis of seven trials by Ebada et al. [50] showed that the efficacy of levetiracetam does not favor its administration in patients with PD for treating levodopa-induced dyskinesia. Finally, we present a summary of the different drugs used to treat motor symptoms in patients with PD in Table 1.

Adenosine A2 receptor antagonists: The FDA recently approved istradefylline as an effective adenosine A2 receptor antagonist that can reduce GABAergic transmission, decreasing the indirect pathway's excitability [46]. It is administered once daily and is available in two formulations, 20 g and 40 g [47]. A previous comparative investigation compared its efficacy to a placebo group and found that it can remarkably reduce the off time by 0.7 [48, 49]. Another study reported a remarkable effect in 40% of patients [47, 48, 50, 51]. Unfortunately, this drug is associated with various adverse events. These include hallucinations, nausea, dyskinesia, and dizziness [46, 47, 52-55].

Gabapentin: The use of gabapentin for managing different PD-related symptoms has been described in the literature. For instance, a previous placebo-controlled, double-blinded, crossover trial showed that the administration of PD significantly enhanced tremors, bradykinesia, and rigidity associated with PD. It should also be noted that the favorable effects of gabapentin on PD-related rigidity and bradykinesia are notable regardless of its impact on tremors [56]. Another trial further showed that the administration of gabapentin significantly enhanced PD-related manifestations, followed by the administration of levodopa, as measured by the basal UPDRS III and the magnitude of the motor response. Although these patients tolerated the drug well, it did not have a noticeable impact on levodopa-induced dyskinesia [57]. Improving peripheral neuropathic pain is another favorable effect gabapentin and pregabalin and has been reported in relevant clinical trials [58, 59]. These trials did not include only PD patients, indicating the need for future investigations [60]. It has been further demonstrated that administering gabapentin and pregabalin can be a secondary option for managing pain in patients with restless syndrome [61].

Safinamide: Its primary mechanism of action is similar to that of MAO-B inhibitors. In addition, it can significantly prevent the formation of free radicals. However, their actions are reversible and selective. It is a benzylamine derivative that can activate voltage-gated sodium channels in a dependent block in the inactivated state [62-66]. Pain-mitigating effects were also reported following the administration of this modality, secondary to its proven anti-glutamatergic activities. The main benefit of safinamide is that it decreases the wearing-off hours without worsening dyskinesia in the advanced stage [27].

Treatments for non-motor manifestations

Many nonmotor manifestations have been reported among PD patients with PD. These include psychosis, depression, dementia, anxiety, dysautonomia (drooling, constipation, and orthostatic hypotension), fatigue, rapid eye movement (REM), sleep disorders, and insomnia [42]. These manifestations can be more disabling than motor manifestations, significantly affecting patients' quality of life with PD. However, the treatment of these conditions is challenging. For instance, psychosis may develop secondary to the administration of pharmacological treatment aimed at reducing motor symptoms. However, changing regimens and dosages of these modalities cannot always be done so that motor manifestations do not worsen. Therefore, different pharmacological treatments have been proposed, including clozapine, olanzapine, quetiapine, risperidone, ziprasidone, pimavanserin, istradefylline, and safinamide. A summary of these therapeutics and their use in patients with PD is presented in Table 2.

**Table 2:** Most commonly prescribed therapeutics for managing Parkinson’s disease-related non-motor symptoms.

Category	Name	Useful for	Additional
Benzodiazepine	Clonazepam/alprazolam/lorazepam/diazepam	Panic, depression, and anxiety	
Tricyclic compounds	Amitriptyline/imipramine/nortriptyline		
Selective serotonin reuptake inhibitors (SSRI)	Sertraline/fluoxetine		Urinary incontinence
Serotonin/norepinephrine reuptake inhibitors (SNRI)	Desvenlafaxin/venlafaxine/duloxetine/milnacipran		
Other anti-anxiety/panic/depression	Buspirone/trazodone/buspirone/quetiapine/mirtazapine/bupropion		
Acetylcholinesterase Inhibitors	Galantamine/donepezil/rivastigmine	Dementia	
Memantine			
Droxidopa/pyridostigmine/fludrocortisone/midodrine		Orthostatic hypotension	
Anticholinergics	Darifenacin/oxybutynin/tolterodine/solifenacin	Overactive bladder + incontinence	
Alpha-1 blockers	Alfuzosin/terazosin/tamsulosin/silodosin		Benign prostatic hyperplasia
Beta-3-agonist	Mirabegron		
Modafinil/Methylphenidate		Daytime sleeping/cognition	
Mirtazapine/doxepin/amitriptyline/trazadone/eszopicione		Insomnia	
Clonazepam			Rapid eye movement sleep behavior disorder
Quetiapine/Pimavanserin/Clozapine		Psychosis/hallucination/delusions	
Polyethylene glycol/Lubiprostone		Constipation	
Trimethobenzamide/Ondansetron		Nausea and vomiting	
Glycopyrrolate/scopolamine patch/atropine drops/botulinum toxin A/botulinum toxin B		Unwarranted drooling	

The efficacy, safety, and mechanism of action of these modalities have been reviewed previously. Among these various modalities, it has been shown that clozapine, pimavanserin, and quetiapine are most commonly used for treating psychosis. However, it should be noted that the worsening of parkinsonism symptoms has been reported with both typical and atypical antipsychotic medications. Moreover, continuous monitoring of patients' vital signs is essential because these medications may be associated with serious adverse events and complications. For instance, screening for agranulocytosis is recommended with clozapine, although it is highly efficacious and clinically useful [42]. Quetiapine is the most commonly used antipsychotic drug. However, reports have shown that it is less efficacious than similar treatment approaches. In addition, the drug is usually associated with antihistaminic effects, leading to drowsiness.

It should be noted that these medications are not recommended in patients with dementia. Treatment of dementia in patients with PD can be effectively performed using NMDA receptor antagonists (memantine), donepezil, and rivastigmine. A previous investigation further demonstrated that the most efficacious treatment modality in this context was rivastigmine [42, 43].

Treating hallucinations might also be challenging in PD patients; for instance, it has been reported that anti-PD drugs can induce psychosis and hallucinations in the following order: most reported cases, anticholinergics, amantadine, MAO-B, dopamine agonists, COMT, and levadopa. In 2016, the FDA approved pimavanserin for the treatment of PD-related delusions and hallucinations [27]. It is administered daily as a 35 mg capsule and acts as a selective serotonin 5-HT2A inverse agonist. Moreover, they do not usually lead to drowsiness because they do not have antihistaminic effects. In another context, anxiety and depression can be treated using cognitive behavioral therapy, selective serotonin-norepinephrine reuptake inhibitors, and selective serotonin reuptake inhibitors. Moreover, it has been reported that using pramipexole is highly efficacious in these situations [42]. Constipation can be treated using different approaches and modalities, such as prebiotic fibers, lubiprostone, laxatives, stool softeners, and probiotics [53].

Furthermore, drowsiness and fatigue can be treated with methylphenidate and modafinil. However, these symptoms are difficult to treat. Insomnia can be treated with quetiapine, mirtazapine, trazodone, hypnotics, and melatonin. Clonazepam and melatonin can also be used to treat REM sleep [53].

Nonpharmacological approaches can be used to manage orthostatic hypotension. Some of these include compression stockings and increased salt and water intake. Gradual changes in position are also encouraged in affected patients. Reducing postprandial hypotension can also be achieved by reducing carbohydrate intake and eating more frequent and smaller meals [45]. Patients should also be encouraged to reduce their alcohol and caffeine consumption because of their diuretic effects [62].

Furthermore, orthostatic hypotension can be worsened by administering certain medications, including dopamine agonists, levodopa, vasodilators, and diuretics. Droxidopa, midodrine, and fludrocortisone can be used to treat orthostatic hypotension. However, physicians should advise PD patients not to lie supine because these medications can significantly lead to supine hypertension. Instead, elevating the head end of the bed by approximately 45° is recommended. Moreover, pressors such as midodrine and droxidopa are not recommended near bedtime [62].

Pimavanserin

Pimavanserin is an inverse agonist or selective antagonist of the 5-hydroxytryptamine (HT)2A receptor. Therefore, it can be effectively used in PD to manage different symptoms [67-70]. Moreover, the drug has reduced affinity for muscarinic, dopaminergic, adrenergic, and histaminergic receptors. Therefore, this drug does not worsen motor symptoms as reported for typical psychotic drugs. Accordingly, the drug was approved by the FDA in 2016 for the treatment of PD-related delusions and hallucinations. Many previous studies have reported the efficacy of pimavanserin in treating PD symptoms [14, 56, 57, 67]. It has also been shown to be effectively used with other drugs, such as quetiapine, enhancing the effectiveness of newly emerging, residual, and resistant PD-related psychotic manifestations [46, 67]. However, some adverse events and complications have been reported with this medication, including hallucinations, peripheral edema, and a confusional state. It is worth mentioning that it is not known whether the drug is safe for lactating or pregnant women. Regarding its effect on the heart, it has been shown that this medication might induce QT interval prolongation, which might contradict its use in cardiac patients [56-58].

Neuroprotective Modalities

Various neuroprotective agents have been reported in the management of PD. Several of these treatments have been validated in clinical trials. The most common neuroprotective agents used are listed in Table 3. Antioxidants include polydatin, melatonin, iron chelators, nicotine, glutathione, and green tea polyphenols. Glucagon-like peptide-1 (GLP-1) has also been examined; it has been shown that GLP-1 mimetics can be used in PD for their potential roles in preserving dopaminergic neurons from degeneration. Intravenous administration of granulocyte colony-stimulating factor (G-CSF) is also under clinical investigation to estimate its motor benefits for patients with PD. Iron chelators might be useful in patients with early PD to reduce oxidative stress, ameliorate cognitive impairment, increase the expression of neurotrophic factors, and reduce striatal dopaminergic neuron loss. Similar protective benefits have also been reported among other neuroprotective agents, and there are ongoing studies investigating further pathways that can help innovate future more potent PD pharmacological treatments (Table 4).

**Table 3:** Currently reported neuroprotective agents that can be used in patients with Parkinson’s disease (PD)

Neuroprotective agent	Outcomes	Mechanism
Pramipexole	No clear benefits	D2/D3 receptor agonist
Ropinirole	Slow the loss of dopamine neurons	D2/D3 receptor agonist
Exenatide	Improve both motor and non-motor functions	Glucagon-like peptide-1 mimetics
Deferiprone	slow disease progression	Iron chelator
Glutathione	No clear benefits	Antioxidant
Green tea polyphenol	No clear benefits	Antioxidant/iron chelation
Isradipine	Improved outcomes	Calcium channel antagonist
Creatine	Safe but no long-term benefits	Ergogenic compound
GPI 1485	Nonfutile	Nonimmunosuppressive immunophilin ligand
Rasagiline/selegiline	Delay the start of PD symptoms	MAO-B inhibitor
Minocycline	Nonfutile, safe, but less tolerable	Anti-inflammatory
Recombinant human erythropoietin (EPO)	Improve both motor and non-motor functions	Anti-inflammatory/antioxidant
Granulocyte-colony stimulating factors	neurogenesis, antiapoptotic, immunity modulation	
Coenzyme Q10	No clear benefits	Antioxidant
N-acetylcysteine	Boost glutathione levels	Antioxidant
Nicotine	Decreases medicine doses and improves motor symptoms	Calcium handline and unfolded protein inhibitor

**Table 4:** Novel pathways that might be relevant for innovating future more potent Parkinson’s disease (PD) therapeutics Adapted from Rai et al. [70]; PTEN, phosphatase and tensin homolog; Nrf2, nuclear factor E2-related factor 2; mTOR, mammalian target of rapamycin; JAK/STAT, Janus kinase/signal transducer and activator of transcription; Erk, extracellular signal-regulated kinase; Akt, protein kinase B.

Novel pathway	Suggested mechanism
PTEN/AKT/mTOR signaling pathway	Impacts PD progression
JAK-STAT signaling pathway	Anti-Parkinson activities were noticed in this pathway after the administration of related therapeutics
Mitochondrial calcium pathway	Reduces neurodegeneration in PD by decreasing related oxidative stress in this pathway
Akt/Nrf2/Erk/ glutathione pathway	A promising pathway that might impact PD progression
Wnt/beta-catenin pathway	This pathway showed promising features in PD progression with some therapeutics

Botulinum Toxin

These modalities have a similar action to anticholinergics, as they inhibit the release of acetylcholine by affecting SNAP and SNARE proteins [38]. The FDA approved four different preparations: rimabotulinum toxin B (Myobloc), incobotulinum toxin A (Xeomin), abobotulinum toxin A (Dysport), and onabotulinum toxin A (Botox). Thus, they can be effectively used to treat various PD-related manifestations. Injection of botulinum toxins into the submandibular and parotid glands can also treat sialorrhea, commonly reported among PD patients, and can lead to aspiration [39, 40]. Similarly, botulinum toxin injection into the detrusor muscle has been approved for managing neurogenic bladder symptoms [57].

Botulinum toxins can treat dystonia, commonly encountered at a young age. Reports also show that it is helpful for striatal limb deformities, peak dose dystonia, off-dystonia, and biphasic dystonia [71, 72]. Some reports have shown that these modalities can treat PD-related camptocormia. However, the reported efficacy in these cases varies [73-76].

Surgical modalities

Various surgical approaches for managing patients with PD have been reported and validated in the literature, although various medications have been validated with favorable efficacies. The importance of these surgical medications is to improve the quality of life of patients with PD who may develop uncontrolled motor fluctuations or prominent tremors. In the present section, we will shed more light on these surgical procedures based on evidence from different studies in the literature. A summary of the surgical modalities is presented in Table 5.

**Table 5:** A summary of surgical treatment modalities for PD

Surgical modality	Technique	Efficacy	Disadvantages
Ablation	Unilateral or bilateral incision of the subthalamic nucleus, thalamus, global pallidus	Highly productive and improves rigidity, tremors, and hypokinesia	Permanent side effects, being irreversible
Focused ultrasound (FUS)	Incision-less, intracranial acoustic energy delivery	Noninvasive, highly efficacious, no permanent side effects	Some adverse events are unilateral only and require conducting other modalities
Levodopa-carbidopa intestinal gel (LCIG)	Levodopa administration through a percutaneous endoscopic gastrostomy	Treats severe motor fluctuations, increases on-time, and reduces off-time	Some adverse events
Deep brain stimulation (DBS)	Stimulation of the basal ganglia by electrical stimuli	Efficacious, safe, treat advances PD, treats motor and non-motor manifestations, long-term efficacy, and reduces off-time	Some adverse events, open loop, less effective for speech symptoms, and balance and gait problems

Radiofrequency Thermocoagulation

Goldman et al. reported in early 1922 that stereotactic ventralis lateralis thalamotomy is an effective treatment for tremors with low surgical risk [77]. In a study comparing radiofrequency thalamotomy, gamma knife thalamotomy, and thalamic stimulation, five out of 13 patients who underwent radiofrequency thalamotomy had an immediate complete response, six had a significant reduction, and two had a partial reduction. Meanwhile, all 11 patients who underwent DBS showed an immediate complete response [78]. However, no long follow-up studies of radiofrequency thalamotomy have been conducted to examine long-term outcomes.

Radiosurgery With Gamma Radiation

In a multicenter study conducted on 72 patients with ET and PD tremors treated with unilateral gamma knife (GK) thalamotomy with a maximum dose of 130 Gy, a good response was observed in 81% of patients, with a recurrence rate of 2.8% after 12 months [79]. Another study evaluated the safety and efficacy of unilateral GK thalamotomy in treating severe tremors with a prospective blinded assessment; improvement in activities of daily living was detected in 72.2% of patients [80].

Magnetic Resonance Imaging-Guided Focused Ultrasound (FUS) Lesioning (Other Targets)

Evidence shows that FUS can be effectively used for intracranial acoustic energy delivery using an incision-less and precise approach. Magnetic resonance thermometry can be used to monitor lesioning. Several studies have assessed the safety and efficacy of this modality. For instance, a previous randomized controlled trial that included 27 patients with PD, of whom 20 received unilateral FUS, showed that the Clinical Rating Scale for Tremor significantly improved in these patients by 62% [81]. However, some adverse events, including ataxia and paresthesia, were reported among these patients, which were permanent in 9% and 14% of the patients, respectively. A similar investigation that included patients with essential tremors also reported similar rates of adverse events [82]. Other reported adverse events included dysarthria, dysphagia, and contralateral weakness. Some intraprocedural adverse events, including dizziness, nausea, and headaches, have also been reported in these patients. However, these events are usually resolved and are not permanent. The risks of gait and speech disturbances during FUS make it difficult to perform bilaterally [83].

The FDA has approved FUS for unilateral thalamotomy in patients with PD. Moreover, thalamotomy has been demonstrated to be efficacious for PD-related tremors and helpful for other PD-related motor symptoms. It has been further reported that akinesia and rigidity can be significantly improved using subthalamotomy [84]. However, this conclusion should be validated and strengthened using evidence from randomized controlled trials. Therefore, it can be concluded that FUS should be used cautiously in patients with PD as a palliative therapy to relieve tremors. However, other surgically invasive procedures can also be used and are discussed subsequently [81].

Levodopa-Carbidopa Intestinal Gel (LCIG)

LCIG is usually conducted using a jejunal extensional tube (P-JET) for percutaneous endoscopic gastrostomy through the administration of the treatment modality. The delivery of levodopa to the small intestine is maintained by an external pump that delivers the drug once per minute [85]. Accordingly, the main aim of this approach is to provide levodopa accurately and avoid bypassing gastric emptying, which maintains a stable concentration of these drugs [86]. The main indication for this approach is that other treatment modalities are insufficient to produce satisfactory outcomes in patients with severe motor fluctuations. The efficacy of this approach has been previously indicated in a meta-analysis, which showed that LCIG increases over time by 0.55 with no troublesome dyskinesia and decreases off-time by 1.19 hours per day in PD patients [87]. Improvements in the Non-Motor Symptom Scale have also been reported following the application of this modality in patients with PD [88, 89]. However, we did not find a relevant randomized controlled trial in the literature that investigated the impact of this outcome on nonmotor symptoms.

Moreover, some adverse events have been reported with the application of LCIG in some studies. These include pump malfunction, abdominal pain, erythema at the stoma, tube obstruction, pneumoperitoneum, peritonitis, infection, and tube dislocation [85]. Moreover, some adverse events have been associated with LCIG infusions, including hallucinations, dyskinesias, and nausea. Some evidence also shows that the procedure may result in peripheral neuropathies [90]. However, the pathophysiology of this event is not well understood. However, it is suggested that vitamin B12 deficiency might be the primary etiology; therefore, it should be regularly monitored [57].

Deep Brain Stimulation (DBS)

DBS can be conducted unilaterally or bilaterally by placing leads into the globus pallidus interna (GPi) or within the subthalamic nucleus (STN) with an implantable pulse generator that might be used in these cases by placing it on the chest of affected patients. The exact mechanism of this approach is not clear in different studies. However, some authors have suggested that it can impact pathological firing patterns in the cortico-basal ganglia network [91, 92]. Uncontrolled tremors, dyskinesias, and motor fluctuations are primary indications for this approach. However, it should be noted that DBS can only relieve symptoms that respond to dopaminergic therapy, except for tremors. Previous studies have compared the efficacy of DBS and pharmacologic treatment and showed that it could significantly increase over time without developing troublesome dyskinesias and decrease medication usage by 50% [93, 94]. Its efficacy also usually extends for up to 10 years [57].

However, some studies have reported that the impact of this approach on rigidity and bradykinesia might be limited to only five years [95, 96]. The timing of DBS remains controversial, as it has been questioned whether DBS should be conducted earlier at the onset of the disease or younger age [57].

In the EARLYSTIM study, a randomized controlled trial, the efficacy of STN DBS among patients with PD at an early stage (mean disease duration and age were 7.5 and 52 years, respectively) was compared to the administration of pharmacological treatment modalities on the impact of early motor complications. The authors demonstrated that the mean quality of life in the STN DBS group significantly improved by 7.8, compared to the standard treatment group, which decreased by 0.2 points. Therefore, it has been concluded that neurostimulation results in more significant outcomes regarding daily living activities, levodopa-induced motor disability and complications, and mobility [97]. Surgical treatment of PD mainly targets the GPi and the STN. Preferring one of these structures over the other is also controversial among the different studies in the literature [98, 99]. The outcomes of both structures were comparable between the two structures, according to a previous randomized controlled trial that used the UPDRS score III to assess the degree of motor improvement [100].

Evidence shows that lowering the need to administer dopaminergic medications is usually associated with subthalamic stimulation. These approaches can replace IPGs because they reduce stimulation parameters, allowing longer stimulation intervals. Moreover, previous studies have compared STN and GPi and found that GPi could be associated with less decline in cognition and mood. Moreover, it has been shown that GPi stimulation can reduce dyskinesias more than STN stimulation. However, dyskinesias might also be reduced with STN owing to the subsequent reduction in medications. Some studies further demonstrated that targeting the thalamic ventral intermediate nucleus (VIM) might also be effective. However, this approach is not commonly used because it is only effective for PD-related tremors and not for other symptoms. In another context, some contraindications may interfere with the use of these surgical modalities. These include unstable psychiatric conditions, symptoms that are unresponsive to levodopa (e.g., gait disturbances), and dementia [57].

Furthermore, some adverse events and complications have been reported following surgical modalities. The rates of these complications are remarkably variable in the literature [101]. For instance, infections have been estimated to be the most commonly reported event, followed by intracranial hemorrhage, lead erosion, and lead migration, with estimated rates of 2-3%, < 2%, 1%, and 1%, respectively. In addition, some adverse events such as cognitive decline, hypomania, depression, gait impairment, diplopia, dysarthria, and paresthesia are related to stimulation [102].

In the current literature, evidence comparing the efficacy of LCIG and DBS has not been adequately established because no previous randomized controlled trials have been conducted. However, a previous meta-analysis demonstrated that the efficacy of DBS and LCIG in enhancing motor function is similar in PD patients [103]. In contrast, a non-randomized open-label investigation showed that, compared to patients receiving STN DBS therapy, conducting LCIG was significantly associated with significant improvement in neuropsychological functions, including visuospatial functions, learning, recognition, and delayed recall [104]. Moreover, the authors reported that STN DBS was not associated with significantly improved behavioral and cognitive functions in patients with PD compared to conventional medical treatment. However, LCIG might be suggested over STN DBS in PD patients, especially when they are older and have severe cognitive functions, due to the potentially reduced risks associated with this approach [57].

Apomorphine Subcutaneous Infusion

The apomorphine subcutaneous pump is an established and effective method for treating PD with motor symptoms that do not respond to oral medications. It was first used to treat PD in 1993 in the United Kingdom (UK). It is known as a high-potency dopamine receptor agonist affecting all dopamine receptor subtypes, reducing off time by up to 80%. Several studies have revealed that apomorphine use can improve Parkinsonian dyskinesia. Studies have shown that the apomorphine pump is the only available medication with an efficacy similar to levodopa. Despite its wide use in clinical approaches to PD, only a limited number of published randomized clinical trials have been conducted to test its efficacy [7, 26, 57].

Emergent Surgical Techniques

Dual-lead DBS: Several studies have demonstrated the utility and effects of emergent surgical techniques. The DBS technique for PD patients has been shown to greatly reduce tremors, which is one of the most critical symptoms affecting patients' lifestyles. The utility and efficacy of DBS in patients with tremors were described by Azghadi et al. Their study demonstrated the effects of DBS on both the globus pallidus interna and the ventral intermediate nucleus of the thalamus in patients who did not achieve intraoperative control of tremors. The data published in this study support the efficacy of DBS in patients with tremors on medication with > 90% tremor reduction [105].

Combining device-aided therapies: DBS, LCIG, and subcutaneous apomorphine infusion are device-aided therapies (DATs) for PD patients. Each should significantly impact the patients, but not for a long time. Studies show that the long-term efficacy is not very high due to the motor and non-motor alterations; however, limited studies have combined more than one DAT in this study. Two such therapies are reported to be optimal for managing PD symptoms. Studies concluded that a personalized combination of interventions in patients with PD could maximize the outcome, but only in some instances, such as (1) persistent symptoms and fluctuation, (2) progression of the disease and reemergence of the motor and non-motor symptoms, (3) severe side effects from the initial therapy, (4) deterioration of patient symptoms, and (5) drug-resistant dysphagia [106].

Many studies reported a substantial improvement in daily OFF time and fluctuations regarding motor outcomes. Regidor et al. reported significant motor improvement assessed by ΟΝ state UPDRS III and axial items of UPDRS following the LCIG addition to DBS, comparable to the control group that switched from oral medications to LCIG monotherapy. Dyskinesias responded well to a second treatment but only in patients with a history of successful treatment of dyskinesias with the first DAT. In conclusion, the axial symptoms did not significantly respond to DAT [106].

Assistive treatment modalities

These modalities can be effectively used to manage hand tremors in PD patients. They are cost-effective and noninvasive, which might favor their use in patients with PD. Various modalities have been proposed in the literature, including devices that can help clinicians establish proper diagnosis and management and others that can help patients [107].

Intelligent Glove

Unintentional trembling can be effectively controlled by wearing an intelligent glove and piezoelectric actuator, which was first designed and reported by Kazi et al. [108]. Hand tremors can also be effectively managed by frequency analysis conducted by an IoT-based device; this was first reported by Ragul et al. [109]. Evidence from further investigations indicates this modality's cost-effectiveness, making it more suitable for patients living in low-income countries [110, 111]. Turkistani et al. [112] further reported the efficacy of sensor-activated wearable gloves that could effectively reduce hand and finger tremors by 40% in patients with PD [108,113,114]. The frequency and severity of hand and finger tremors can be effectively measured using a wearable wristwatch, as suggested by Jeon et al. [115].

Adaptive Tremor Cancellation

Detecting hand tremors can also be performed using an adaptive control system, including surface Electromyography (EMG), which has been validated in previous studies [56, 110, 111]. Papini et al. [116] validated the use of a wearable motion tracker that can effectively suppress hand tremors.

Cantilever Vibration Control 

Other similar modalities aimed at suppressing hand tremors, such as tuned vibration absorbers, adaptive tremor cancellation technology, signal sensors, and cantilever Vibration Control Other devices, have been validated in the literature with favorable outcomes in patients with PD [107].

Palliative approaches

Although surgical and pharmacological treatment modalities might be effective in treating patients with PD, evidence shows that these modalities cannot adequately control some related symptoms. As previously discussed, the efficacy of previous modalities might be limited by associated adverse events and complications. Some PD-related symptoms, such as gait disorders, including stride variability, freezing of gait, balance problems, and postural instability, have been reported in the literature. Accordingly, physicians should consider other treatment regimens to improve these symptoms, which might impact patients' quality of life with PD. Different palliative therapeutics, including speech and physical therapy, have been proposed to manage the motor symptoms associated with PD. Some authors have reported the efficacy of occupational therapy, mainly designed to enable patients with PD to pursue their daily activities and be involved in the community. These therapeutics are focused on the patient’s behavior, aiming at symptomatic management instead of modifying or supplementing the affected neuronal pathways [84-88]. These modalities are summarized in Table 6.

**Table 6:** A summary of available palliative treatment modalities for PD

Treatment	Useful for	Efficacy	Disadvantages
Physical therapy	Motor-related symptoms	Various forms exist, allowing for patient comfort, enhancing the quality of life, and treating multiple symptoms	Needs patient compliance and involvement of other modalities
Speech therapy	Voice exercise	Improves the quality of life and improves multiple voice symptoms	Limited to speech symptoms
Occupational therapy	Maintains pursuing daily activities	Improves self-confidence and social involvement, and promotes independence	It needs compliance, is not helpful with psychological symptoms, and requires other modalities, like physical therapy
Cognitive behavioral therapy	Behavioral training for managing symptoms	It can reduce symptoms and dispense with medications and related complications, like insomnia, and can be remotely conducted	Patient compliance needs testing, effective for one symptom, and cannot be tested in a double-blinded manner

Limitations

The data referenced in this study have some limitations, as there was variation in the management approaches of each individual in those studies. The research was gathered from multiple databases such as PubMed, Google Scholar, and others after applying appropriate filters; however, some of these databases may miss relevant publications. Furthermore, some data relevant to PD management may have been missed, as some research may have been published during the writing of this paper.

## Conclusions

This paper reviews the literature on PD management and summarizes the progress made in medical, surgical, and assisted therapeutic modalities for motor and non-motor symptoms. PD is a progressive neurological disorder arising from a decrease in dopamine production due to dopaminergic neuronal death in the substantia nigra, leading to disruption of the nigrostriatal system. PD is a clinical diagnosis based on the patient’s clinical features and the exclusion of other possible causes of parkinsonism. PD management requires individual customization based on disease stage and progression.

The management options and assisted methodologies of PD are improving, with new therapeutic options emerging every year. Medical therapy is the most common therapy as it corrects dopamine deficiency, which is the main factor implicated in PD, and other surgical treatment options are available in cases of a chronic progressive disease course.
